# Clinical phenotypes, aetiologies, management, and mortality in acute heart failure: a single‐institution study in Latin‐America

**DOI:** 10.1002/ehf2.13092

**Published:** 2020-11-11

**Authors:** Héctor González‐Pacheco, Amada Álvarez‐Sangabriel, Carlos Martínez‐Sánchez, José L. Briseño‐Cruz, Alfredo Altamirano‐Castillo, Salvador Mendoza‐García, Daniel Manzur‐Sandoval, Luis M. Amezcua‐Guerra, Julio Sandoval, Rafael Bojalil, Diego Araiza‐Garaygordobil, Daniel Sierra‐Lara, Carlos A. Guiza‐Sánchez, Rodrigo Gopar‐Nieto, Camelia Cruz‐Rodríguez, José J. Valdivia‐Nuño, Brandon Salas‐Teles, Alexandra Arias‐Mendoza

**Affiliations:** ^1^ Coronary Care Unit National Institute of Cardiology in Mexico City Juan Badiano, Sección XVI, Tlalpan Mexico City 14080 Mexico; ^2^ Heart Failure Clinic and Transplantation National Institute of Cardiology in Mexico City Mexico City Mexico; ^3^ Department of Immunology National Institute of Cardiology in Mexico City Mexico City Mexico

**Keywords:** Acute heart failure, Clinical phenotypes, Latin America, Mortality

## Abstract

**Aims:**

Little is known regarding acute heart failure (AHF) clinical characteristics and its hospital outcome in Latin America. This study sought to assess the prevalence of, and identify differences among, in‐hospital outcomes in patients hospitalized for AHF who were stratified by clinical phenotype at a hospital in Latin America.

**Methods and results:**

This is a retrospective cohort study of patients with AHF who were hospitalized in the coronary care unit of a Latin American teaching hospital from January 2006 to December 2018. Cox regression analysis was used to identify predictors of mortality. Of 21 042 patients admitted, 7759 (36.6%) had AHF. Their median age was 62 years, and 35% were women. *De novo* heart failure was seen in 39.4% of patients. Most common was AHF‐associated acute coronary syndromes (ACS‐HF) in 43.0%, decompensated heart failure (DHF) in 33.7%, hypertensive heart failure (HT‐HF) in 11.8%, and cardiogenic shock (CS) in 5.2%. Pulmonary oedema (PO) (3.3%) and right heart failure (RHF) (3.0%) were least frequent. Coronary artery disease was the most frequent aetiology in 56.5% of patients, valvular heart disease in 22.4%, and cardiomyopathies in 12.3%. Other less frequent aetiology included adult congenital heart disease (2.5%), lung diseases (2.1%), acute aortic syndromes (1.4%), pericardial diseases (0.8%), and intracardiac tumours (0.3%). Aetiology could not be established in 1.6% of patients. Before admission, patients with worsening chronic heart failure and reduced ejection fraction were treated with renin–angiotensin system blockers (60.4%), beta‐blockers (42.5%), or spironolactone (34.4%). The percentages of patients given in‐hospital management with intravenous diuretics, vasodilators, inotropes, and vasopressors were 81.2%, 33.4%, 18.9%, and 20.4%, respectively. The overall in‐hospital mortality was 17.9% (71.3%, 43.9%, 23.8%, 14.9%, 13.6%, and 10.1% for CS, PO, RHF, DHF, ACS‐HF, and HT‐HF, respectively; *P* < 0.0001). Multivariate analysis revealed that PO (hazard ratio [HR] 2.68, 95% confidence interval [CI] 1.73–4.14, *P* < 0.0001) and CS (HR 3.37, 95% CI 2.12–5.35, *P* < 0.0001) were independent predictors of in‐hospital mortality. Use of intravenous diuretics was linked to reduction of in‐hospital mortality (HR 0.70, 95% CI 0.59–0.59, *P* < 0.0001). By contrast, increased in‐hospital mortality was associated with the use of intravenous inotrope or vasopressor (HR 1.49, 95% CI 1.27–1.76 and HR 2.91, 95% CI 2.41–3.51, *P* < 0.0001, respectively).

**Conclusions:**

Real‐world evidence from a university hospital in Latin America shows that the high mortality among patients with AHF may depend, among other factors, on patients' AHF clinical phenotypes. The clinical characteristics and aetiologies of AHF appear to differ between these data from Mexico and those from European and US registries.

## Introduction

It is well known that in Europe and the USA, acute heart failure (AHF) is the leading cause of hospitalizations and that its high morbidity, mortality, and economic burden make it an important public health issue.^1^, ^2^ AHF is a heterogeneous and haemodynamically diverse syndrome that is frequently life‐threatening and requires urgent therapy. Accordingly, current recommendations emphasize the importance of immediate diagnosis and treatment of patients presenting with AHF. Several classification schemes have been proposed.^3^ However, because clinical presentation at admission is highly heterogeneous, it may be more prudent to stratify patients with AHF based on their initial clinical presentation. In 2008, the European Society of Cardiology (ESC) proposed that patients be classified according to six clinical profiles at initial presentation: decompensated chronic HF (DHF), pulmonary oedema (PO), hypertensive HF (HT‐HF), cardiogenic shock (CS), right HF (RHF), and HF in the setting of acute coronary syndromes (ACS‐HF).^4^

The primary data available on patients with AHF come from several large‐scale registries developed in Europe^5^, ^6^, ^7^ and the USA,^8^ although hospital‐based registries remain the primary source of real‐world data about AHF.^9^ The roles of geographic differences and income inequality may be related to differences in patient characteristics, outcomes, and, most importantly, the effects of treatment observed in HF trials.^10^ Because information on the prevalence of clinical phenotypes, management, and hospital outcomes of patients admitted for AHF in Latin America is scarce, we conducted a retrospective analysis to gain insight into the prevalence of different clinical presentation phenotypes as well as the aetiology, treatment, and hospital outcomes of AHF patients admitted to a contemporary teaching hospital in Mexico City.

## Materials and methods

This was a retrospective cohort study using the database of the coronary care unit (CCU) of the National Institute of Cardiology in Mexico City. We analysed data from all consecutive patients admitted to the CCU between 1 January 2006 and 31 December 2018 with a diagnosis with AHF.

We gathered demographic characteristics, medical history, physiological parameters at admission (i.e. blood pressure and heart rate), biochemical findings, in‐hospital treatments, and in‐hospital mortality. In‐hospital mortality was defined as all‐cause mortality during hospitalization. Vital signs were determined at the initial medical presentation. Baseline creatinine clearance was estimated using the Cockcroft–Gault formula. We included patients with any type of AHF (i.e. including *de novo* or worsening chronic HF [WCHF]) at the time of presentation. *De novo* AHF was defined as AHF in patients with no prior history of HF. WCHF was defined as worsening of HF in patients with a previous diagnosis with, or hospitalization for, HF.

Because patients were not previously classified into one of the six AHF clinical phenotypes, we classified them at the time of admission based on the 2008 ESC guidelines^4^: (i) DHF—a history of progressive worsening of chronic HF with treatment and evidence of systemic and pulmonary congestion; does not fulfil criteria for CS, PO, or hypertensive crisis; (ii) HT‐HF—signs and symptoms of AHF are accompanied by high blood pressure (systolic blood pressure ≥ 140 mmHg) at admission; (iii) PO—symptoms of AHF are accompanied by severe respiratory distress, orthopnea, and crackles over the lungs; (iv) CS—we used the clinical definition of CS from the IABP‐SHOCK II study^11^ with clinical criteria of systolic blood pressure < 90 mmHg for ≥30 min or catecholamines to maintain systolic blood pressure > 90 mmHg and clinical pulmonary congestion and lactate > 2.0 mmol/L; (v) RHF—evidence of right ventricular dysfunction and signs of systemic congestion; and (vi) ACS‐HF—signs and symptoms of HF in the presence of ACS, with the ACS diagnosis based on clinical characteristics, electrocardiographic changes, and biochemical markers of cardiac necrosis (creatinine kinase isoenzymes, creatinine phospho‐kinase, or troponin I above the upper normal limit).

The following primary underlying cardiopulmonary aetiologies were documented: (i) coronary artery disease (including ACS and ischaemic cardiomyopathy); (ii) valvular heart disease (aetiology organic including endocarditis and valvular prosthesis dysfunction); (iii) cardiomyopathies (including idiopathic dilated, hypertrophic, diabetic, hypertensive, peripartum, left ventricular non‐compaction, chagasic, restrictive, and Takotsubo cardiomyopathies, and myocarditis); (iv) lung diseases (e.g. pulmonary embolism, pre‐existing lung disease, and arterial pulmonary hypertension); (v) pericardial disease; (vi) intracardiac tumours; (vii) adult congenital heart disease; and (viii) acute aortic syndromes.

Additionally, depending on the left ventricular ejection fraction (LVEF) recorded by echocardiography during hospitalization, patients were categorized into LVEF subgroups, defined as <40%, 40–49%, and ≥50%, based on the 2016 ESC guidelines.^3^

## Statistical analyses

Continuous variables were tested for normal distribution using the Kolmogorov–Smirnov test and are presented as medians and the 25th and 75th percentiles (interquartile ranges [IQRs]). Categorical variables are reported as values and percentages. Differences in baseline characteristics across clinical phenotype categories at study entry were assessed with either χ^2^ or Fisher's exact probability tests, for categorical variables, or the Kruskal–Wallis test for continuous variables.

The primary study outcome was all‐cause in‐hospital mortality. In‐hospital mortality rates were calculated for each clinical phenotype and expressed as a percentage; group differences were evaluated by χ^2^ tests. Using the cohort of patients admitted during the same period without HF as a reference, differences in mortality between the AHF phenotypes were investigated, survival was plotted with the Kaplan–Meier curve, the patients were censored at hospital discharge, and differences between groups were assessed by a log‐rank test. An age‐adjusted and gender‐adjusted Cox proportional hazards regression model was used to estimate the association between each clinical phenotype and their in‐hospital risks of death, compared with patients without HF. Univariate and multivariate Cox's proportional hazards regression models with backward selection were used to identify significant predictors of in‐hospital all‐cause mortality. Two multivariable analyses were performed using the two models. Model 1 included only variables available at the time of admission (demographic variables, medical history, clinical features on presentation, laboratory data—except N‐terminal pro‐brain natriuretic peptide [NT pro‐BNP] levels, which were missing for 30% of patients—and clinical phenotypes) that were associated (*P* < 0.05) with mortality in the univariate analysis.

Model 2 was used to assess the potential role of in‐hospital management in AHF patients. We repeated the multivariate model that included the use of intravenous diuretics, inotropes, vasopressors, intra‐aortic balloon pump (IABP), and mechanical ventilation. The hazard ratio (HR) with 95% confidence interval (CI) was calculated. All tests were two‐sided, and *P* < 0.05 was considered statistically significant. IBM SPSS Statistics for Windows, version 23 (IBM Corp., Armonk, NY, USA) was used.

## Results

### Baseline characteristics of the study population

During the study period (January 2006 to December 2018), 21 042 consecutive patients were admitted to the CCU. Among these, overall AHF was documented in 7759 (36.6%). Of all patients with AHF, 3058 (39.4%) presented with *de novo* AHF, and WCHF was diagnosed in 4701 (60.6%).

**Figure 1:**
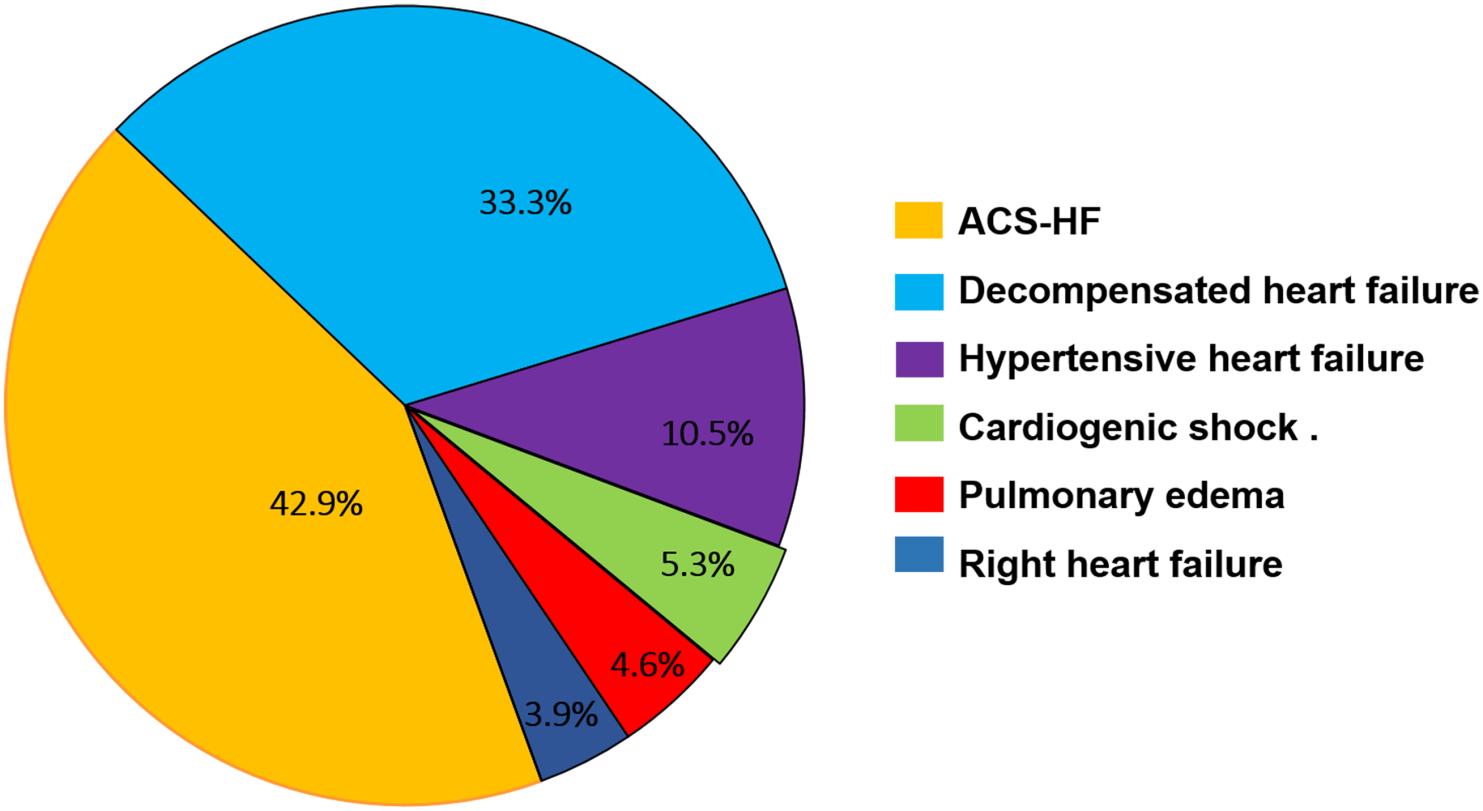
Classification of acute heart failure patients by clinical profile.

The study sample was classified based on ESC clinical profile guidelines, and the prevalence rate of each was calculated. ACS‐HF, making up 3338 (43.0%) of all cases, was the most common clinical class at admission, followed by DHF in 2617 (33.7%), HT‐HF in 914 (11.8%), and CS in 404 (5.2%). Least frequent were PO, present in 255 (3.3%), and RHF, in 231 (3.0%) (*Figure* *1*). Patient baseline characteristics in each clinical phenotype category are shown in *Table* *1*. Among the whole sample, the median age was 62 years (IQR 52–72 years), 35.0% were women, and there were high rates of a history of hypertension (53.5%), diabetes (37.0%), smoking (47.9), HF (60.6%), and previous myocardial infarction (23.2%). Patients with HT‐HF and ACS‐HF were older, while the proportion of women was highest in RHF, HT‐HF, and PO. Comorbidities such as diabetes were more frequent among patients with ACS‐HF or CS, hypertension was more frequent among those with HT‐HF and ACS‐HF, and a previous history of HF was more frequent among those with DHF, HT‐HF, or PO.

**TABLE 1:** Comparison of baseline characteristics of patients according to the clinical phenotypes

	Overall (*n* = 7759)	ACS‐HF (*n* = 3338)	DHF (*n* = 2617)	HT‐HF (*n* = 914)	CS (*n* = 404)	PO (*n* = 255)	RHF (*n* = 231)	*P* value
Age median (IQR) (years)	62 (52–72)	63 (56–72)	60 (47–71)	65 (54–75)	62 (52–72)	54 (41–66)	49 (32–64)	<0.0001
Female (%)	35.0	24.5	41.3	47.4	35.4	40.4	59.3	<0.0001
Body mass index median (IQR) (kg/m^2^)	26.1 (23.7–29.0)	26.6 (24.2–29.2)	25.3 (22.9–28.1)	26.8 (24.0–30.4)	26.1 (23.8–29.2)	25.3 (22.8–27.6)	26.4 (22.9–30.2)	<0.0001
Medical history								
Current smoking (%)	16.7	25.0	8.7	9.7	23.8	11.4	7.4	<0.0001
Previous smoking (%)	31.2	34.2	30.5	28.6	25.2	28.6	20.3	<0.0001
Hypertension (%)	53.5	60.6	41.7	74.6	49.3	32.9	31.2	<0.0001
Dyslipidaemia (%)	27.0	37.3	17.6	25.3	25.0	11.8	10.0	<0.0001
Diabetes (%)	37.0	51.1	22.7	37.6	42.1	12.9	11.7	<0.0001
Previous heart failure (%)	60.6	28.5	100.0	73.0	33.4	75.7	59.7	<0.0001
Previous MI (%)	23.2	32–0	16.9	19.7	18.3	11.4	3.5	<0.0001
Previous PCI (%)	9.9	13.5	7.1	9.0	8.2	5.1	0.9	<0.0001
Previous CABG (%)	4.1	4.7	4.2	3.9	2.2	2.7	1.0	0.01
Previous valvular surgery (%)	8.4	0.9	16.5	9.3	9.7	21.6	5.6	<0.0001
Previous stroke (%)	5.2	4.0	6.5	5.4	6.9	8.6	1.7	<0.0001
Previous AF (%)	16.2	3.7	28.6	21.7	16.8	30.2	18.6	<0.0001
Clinical presentation								
*De novo* AHF (%)	39.4	71.5		27.0	66.6	24.3	40.3	<0.0001
WCHF (%)	60.6	28.5	100.0	73.0	33.4	75.7	59.7	<0.0001
SBP median (IQR) (mmHg)	130 (104–139)	125 (110–140)	110 (100–121)	155 (145–174)	80 (70–85)	105 (90–117)	110 (100–120)	<0.0001
HR median (IQR) (beats/min)	90 (75–104)	85 (74–100)	90 (75–108)	94 (78–110)	100 (65–113)	100 (80–120)	96 (80–110)	<0.0001
Peripheral oedema (%)	39.6	15.7	59.7	60.0	31.7	68.2	56.7	<0.0001
Pulmonary congestion (%)	81.4	84.9	79.4	80.2	84.2	89.4	42.9	<0.0001
Laboratory values								
Haemoglobin, median (IQR) (g/dL) (*n* = 7744)	13.7 (11.8–15.2)	14.0 (12.3–15.5)	13.2 (11.3–14.9)	13.45 (11.3–15.0)	13.9 (11.9–15.8)	12.9 (11.3–14.5)	14.2 (11.7–16.1)	<0.0001
Sodium, median (IQR) (mEq/L) (*n* = 7747)	136 (133–139)	137 (134–139)	136 (132–138)	137 (134–140)	136 (132–139)	134 (130–138)	136 (133–139)	<0.0001
Sodium < 136 mEq/L (%) (*n* = 7747)	42.6	38.3	49.1	31.9	49.6	60.2	42.2	<0.0001
Albumin, median (IQR) (g/dL) (*n* = 7235 patients)	3.5 (3.1–3.8)	3.5 (3.2–3.8)	3.4 (3.0–3.8)	3.4 (3.0–3.8)	3.2 (2.8–3.5)	3.3 (3.0–3.6)	3.2 (2.8–3.6)	<0.0001
Albumin, <3.5 g/dL (*n* = 7235 patients)	49.1	42.3	51.7	52.7	69.6	65.7	64.4	<0.0001
hs‐CRP, median (IQR) (mg/L) (*n* = 7099 patients)	22.1 (8.0–73.6)	25.0 (8.0–87.0)	18.0 (7.0–57.0)	16.6 (7.2–49.7)	59.8 (23.5–146.5)	38.5 (14.3–102.2)	26.8 (10.2–79.2)	<0.0001
NT‐proBNP (pg/mL), median (IQR) (*n* = 5657 patients)	5540 (2213–15 506)	4000 (1857–9043)	7188 (2624–19 204)	7107 (2340–21 257)	15 112 (5251–25 000)	16 450 (5115–25 000)	4627 (1874–13 737)	<0.0001
eGFR, median (IQR) (mL/min) (*n* = 7726)	59.0 (36.2–85.1)	65.4 (41.8–90.1)	56.6 (35.0–81.8)	52.5 (32.1–78.1)	36.6 (25.2–56.6)	52.9 (32.6–81.2)	70.9 (45.3–100.5)	<0.0001
eGFR, ≤30 mL/min (%) (*n* = 7726)	18.2	14.0	19.9	22.7	34.5	20.2	11.8	<0.0001
ECHO available (*n*)	7459	3225	2536	882	349	245	222	
LVEF median (IQR) (%)	40 (30–50)	40 (32–46)	40 (27–55)	45 (32–58)	30 (23–41)	35 (25–55)	59 (50.7–63)	<0.0001
LVEF < 40% (%)	46.1	44.9	49.3	38.0	70.2	58.4	7.2	<0.0001
LVEF 40–49% (%)	24.8	34.7	18.3	20.9	11.7	9.8	7.2	<0.0001
LVEF ≥ 50% (%)	29.1	20.3	32.4	41.2	18.1	31.8	85.6	<0.0001
Aetiology								
Coronary artery disease (%)	56.5	100.0	19.6	23.6	69.1	12.9	3.5	<0.0001
Valvular heart disease (%)	22.4	0	46.2	31.3	15.6	60.0	10.8	<0.0001
Cardiomyopathies (%)	12.3	0	22.7	28.7	10.9	19.6	2.6	<0.0001
Adult congenital heart disease (%)	2.5	0	3.2	2.2	1.0	3.1	33.3	<0.0001
Lung diseases (%)	2.1	0	1.1	3.0	2.0	0	44.2	<0.0001
Acute aortic syndromes (%)	1.4	0	2.6	3.7	0.2	1.2	0.9	<0.0001
Pericardial diseases (%)	0.8	0	1.5	1.5	0.2	1.6	1.7	<0.0001
Intracardiac tumours (%)	0.3	0	0.6	0.4	0.2	0	2.2	<0.0001
Aetiology not established (%)	1.6	0	2.4	5.6	0.7	1.6	0.9	<0.0001

ACS‐HF, acute heart failure and associated acute coronary syndromes; AF, atrial fibrillation; AHF, acute heart failure; CABG, coronary artery bypass grafting; CS, cardiogenic shock; DHF, decompensated heart failure; ECHO, echocardiography; eGFR, estimated glomerular filtration rate (according to the Cockroft–Gault formula); HR, heart rate; hs‐CRP, high‐sensitivity C‐reactive protein; HT‐HF, hypertensive heart failure; LVEF, left ventricular ejection fraction; MI, myocardial infarction; NT‐proBNP, N‐terminal pro‐brain natriuretic peptide; PCI, percutaneous coronary intervention; PO, pulmonary oedema; RHF, right heart failure; SBP, systolic blood pressure; WCHF, worsening chronic heart failure.

### Clinical presentation

*De novo* AHF was more frequent in patients with ACS‐HF, and WCHF was more frequent in those with DHF. At presentation, as expected, patients with CS were more likely to have high‐risk features, including lower systolic blood pressure, higher heart rate, lower LVEF, and lower estimated glomerular filtration rates (eGFRs) (*P* < 0.0001) (*Table* *1*). Peripheral oedema and pulmonary congestion were found in 39.6% and 81.2% of patients, respectively, and were more frequently observed in patients admitted with PO or DHF.

Overall, sodium levels < 136 mEq/L and albumin < 3.5 g/dL were observed in 42.5% and 49.1% of patients, respectively. Hyponatraemia (sodium levels < 136 mEq/L) was more frequent in patients with PO, DHF, or CS, while hypoalbuminaemia (albumin < 3.5 g/dL) was more frequent in patients with CS, RHF, or PO. In contrast, levels of high‐sensitivity C‐reactive protein and NT pro‐BNP were significantly elevated in all clinical profiles, especially in patients with CS or PO.

The median estimated eGFR of all patients was 59.0 mL/min, with the best renal function in those with RHF or ACS‐HF. Among the 18.2% of patients with eGFR ≤ 30 mL/min, the CS group (33.9%) was primarily represented.

Most patients (96.1%) underwent echocardiography during hospitalization. Overall, their median LVEF was 40% (IQR 30–50). Left ventricular function also differed markedly among the clinical profiles, varying from 30% (IQR 23–41) in CS patients to 59% (IQR 50.7–63) in RHF patients. Reduced left ventricular function (LVEF < 40%) was present in 46.1% of the overall sample and was more common in CS patients (70.2%). In contrast, preserved left ventricular function (LVEF ≥ 50%) was present in 29.1% of the overall sample and was more common in RHF (85.6%) and HT‐HF (41.2%) patients.

### Major acute heart failure aetiology

**Figure 2:**
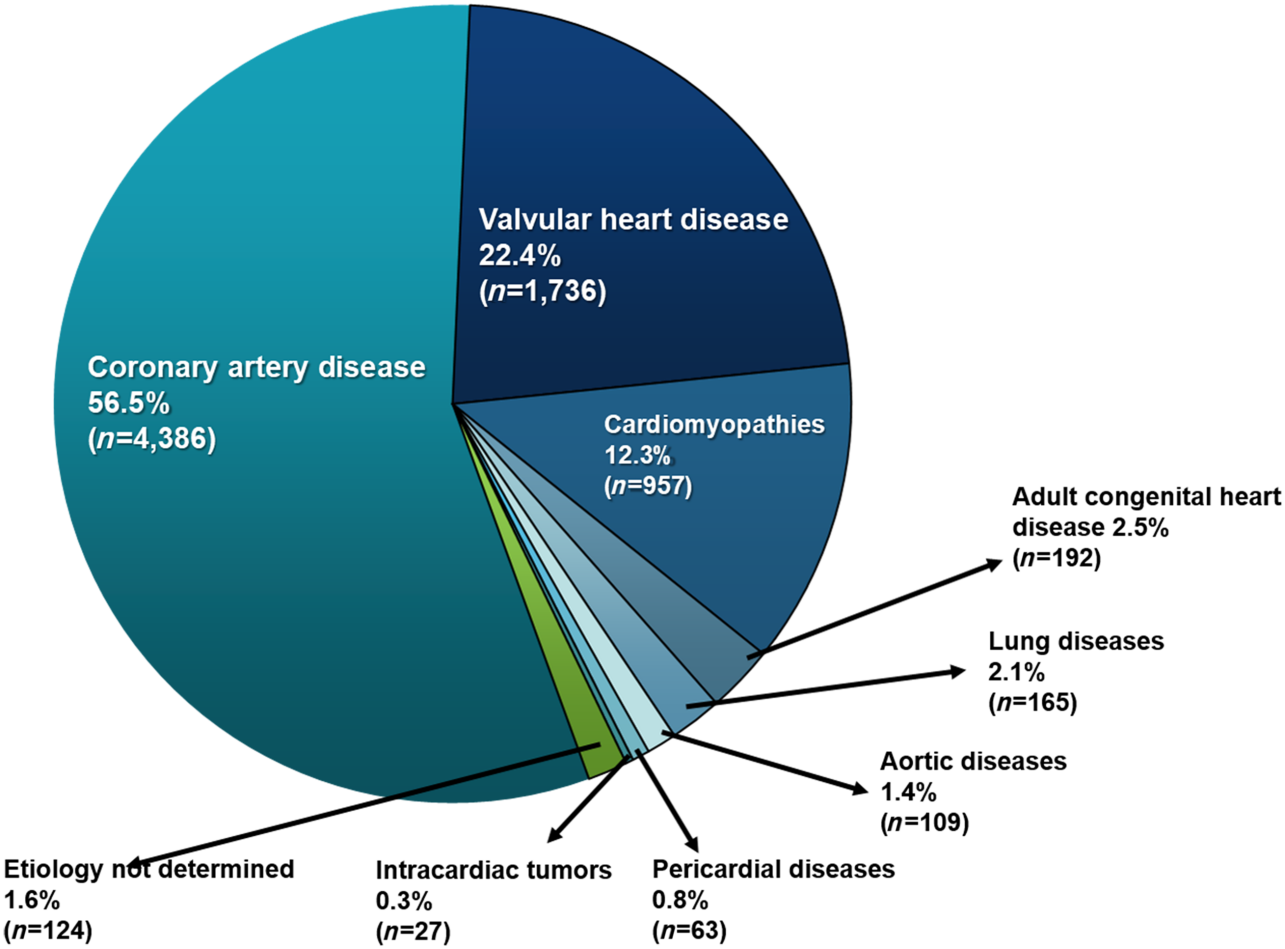
Aetiologies of acute heart failure. *Coronary artery disease included ACS (*n* = 3597) and chronic ischaemic cardiomyopathy (*n* = 789). **Cardiomyopathies included idiopathic dilated (*n* = 510), hypertensive (*n* = 237), chagasic (*n* = 56), hypertrophic (*n* = 37), restrictive (*n* = 27), peripartum (*n* = 17), left ventricular non‐compaction (*n* = 10), Takotsubo (*n* = 12), diabetic (*n* = 6), and myocarditis (*n* = 45).

In our sample, coronary artery disease and valvular heart disease were the most frequent underlying aetiologies, observed in 4386 (56.5%) and 1736 (22.4%) of cases, respectively, followed by cardiomyopathies in 957 (12.3%), adult congenital heart disease in 192 (2.5%), lung diseases in 165 (2.1%), acute aortic syndromes in 109 (1.4%), pericardial diseases in 63 (0.8%), and intracardiac tumours in 27 (0.3%). Aetiology could not be established for 124 (1.6%) of the cases (*Figure* *2*). The differences in aetiologies between patients with varying clinical phenotypes are shown in *Table* *1*. Of the 404 patients with CS, coronary artery disease (i.e. ACS) was the most frequent underlying disease, observed in 279 (69.1%) of patients; valvular heart disease was the main aetiology in patients with PO (60.0%), DHF (46.2%), and HT‐HF (31.3%).

### Medical history prior to admission

Data about treatment prior to admission were available in 94.8% (7355/7759) of patients. Previous use of all analysed drugs was more frequent in patients with WCHF compared with those with *de novo* HF (Supporting Information, *Table* *S1*). In the whole sample with WCHF, before hospitalization, more than half received angiotensin‐converting enzyme inhibitors (ACEIs) or angiotensin receptor blockers (ARBs) (55.4%) and diuretics (54.2%); these were more frequently used by patients in the ACS‐HF, CS, or HT‐HF groups and by those in the DHF, PO, or CS groups, respectively. Previous use of beta‐blockers occurred among 38.4% of patients, and their use was more frequent in patients with CS or ACS‐HF. Digoxin and spironolactone were used by approximately one‐third of patients with WCHF.

Among the WCHF patients with LVEF < 40%, the ACEIs/ARBs, beta‐blockers, and spironolactone were used in 60.4%, 42.5%, and 34.4%, respectively. Surprisingly, only 32.8% of these patients were treated before admission with ACEIs or ARBs plus beta‐blockers. Up to 25% of patients did not previously receive ACEIs/ARBs, beta‐blockers, or mineralocorticoid receptor antagonists (*Table* *2*).

**TABLE 2:** Medications at hospital admission in worsening chronic heart failure according to left ventricular ejection fraction (available data in 4268 patients)

	Overall (*n* = 4268)	LVEF < 40% (*n* = 2254)	LVEF 40–49% (*n* = 864)	LVEF ≥ 50 (*n* = 1150)	*P* value
ACEI (%)	55.6	51.0	40.5	41.6	<0.0001
ARBs (%)	13.5	13.2	14.0	10.7	0.83
ACEI or ARBs (%)	55.6	60.4	57.3	44.7	<0.0001
Beta‐blocker (%)	38.5	42.5	37.4	31.3	<0.0001
Diuretics (%)	54.4	58.0	50.9	49.9	<0.0001
Spironolactone (%)	28.8	34.4	22.7	22.3	<0.0001
ACE/ARBs + beta‐blockers + spironolactone (%)	11.4	16.0	7.9	5.0	<0.0001
ACE/ARBs + beta‐blockers (%)	27.3	32.8	26.9	16.9	<0.0001
ACE/ARBs + spironolactone (%)	19.9	25.4	14.0	13.6	<0.0001
None of the previous three (%)	27.8	25.0	26.3	34.5	<0.0001

ACEIs, angiotensin‐converting enzyme inhibitors; ARBs, angiotensin receptor blockers; LVEF, left ventricular ejection fraction.

### In‐hospital management

In‐hospital management is reported in *Table* *3*. Intravenous diuretics were used in most patients (81.2%), particularly those with HT‐HF, DHF, or PO. Overall, the use of inotropes and vasopressors in patients was 18.9% and 20.4%, respectively; as expected, their use in patients with CS was more frequent (inotropes 72.0%, vasopressors 96.3%), as were the use of IABP and mechanical ventilation. Among inotropes, dobutamine was used more frequently, while intravenous vasodilators were used in approximately one‐third of patients, primarily those with ACS‐HF, HT‐HF, or PO.

**TABLE 3:** In‐hospital management according to the clinical phenotypes

	Overall (*n* = 7759)	ACS‐HF (*n* = 3338)	DHF (*n* = 2617)	HT‐HF (*n* = 914)	CS (*n* = 404)	PO (*n* = 255)	RHF (*n* = 231)	*P* value
Intravenous diuretics (%)	81.2	76.6	86.9	88.9	65.6	85.1	71.4	<0.0001
Intravenous vasodilators any (%)	33.4	43.8	21.5	49.3	5.4	28.6	9.1	<0.0001
Nitroglycerine (%)	30.9	43.4	17.7	43.4	5.4	20.0	8.7	<0.0001
Nitroprusside (%)	3.1	0.9	4.3	7.7	0.5	9.4	0.4	<0.0001
Intravenous diuretic + vasodilators (%)	29.0	35.4	20.6	45.8	5.0	28.2	7.8	<0.0001
Inotropes any (%)	18.9	15.3	16.7	8.9	72.0	36.5	21.6	<0.0001
Dobutamine (%)	15.6	13.5	12.5	5.8	64.9	31.0	18.6	<0.0001
Levosimendan (%)	2.9	2.7	2.5	0.8	11.9	5.5	1.3	<0.0001
Dopamine (%)	4.5	2.5	5.7	3.5	13.4	9.0	5.2	<0.0001
Vasopressors any (%)	20.4	16.1	15.6	7.4	96.3	46.3	28.1	<0.0001
Norepinephrine (%)	20.1	15.8	15.3	7.2	95.8	43.9	26.8	<0.0001
Vasopressin (%)	10.1	7.6	5.6	3.5	63.1	24.7	13.4	<0.0001
Both vasopressors (%)	9.7	7.3	5.3	3.3	62.6	22.4	12.1	<0.0001
ACEI (%)	64.9	79.8	57.6	66.8	21.8	37.6	31.2	<0.0001
ARB (%)	3.0	1.8	3.7	6.6	1.0	1.6	2.2	<0.0001
ACEI or ARB (%)	66.9	80.8	60.3	70.7	22.8	38.8	32.9	<0.0001
Beta‐blocker (%)	32.0	48.6	21.2	23.2	11.4	12.2	8.7	<0.0001
Spironolactone (%)	19.1	17.7	24.2	15.8	10.6	17.3	13.0	<0.0001
Digoxin (%)	19.8	7.9	31.8	25.5	15.6	34.9	22.5	<0.0001
Amioradone (%)	12.0	10.4	13.3	10.1	21.8	12.9	10.0	<0.0001
IABP (%)	5.1	8.0	0.8	0.3	25.7	2.0	0.0	<0.0001
Mechanical ventilation (%)	14.7	12.0	8.9	10.0	66.8	39.6	19.5	<0.0001
Coronary angiography (%)	43.2	75.2	13.3	17.8	46.5	13.7	3.0	<0.0001
Primary PCI (%) (*n* = 2035, STEMI patients)^a^	24.9	24.7			26.0			
Thrombolysis in our hospital (%)	2.9	3.1			1.3			
Thrombolysis outside our hospital (%)	19.2	19.2			19.3			

ACEIs, angiotensin‐converting enzyme inhibitors; ACS‐HF, acute coronary syndrome and HF; ARBs, angiotensin receptor blockers; CS, cardiogenic shock; DHF, decompensation heart failure; HT‐HF, hypertensive HF; IABP, intra‐aortic balloon pump; PCI, percutaneous coronary intervention; PO, pulmonary oedema; RHF, isolated right HF; STEMI, ST‐elevation myocardial infarction.

^a^ STEMI: ACS‐HF = *n* = 1812 and CS = *n* = 223.

Overall, ACEIs or ARBs were used in approximately two‐thirds of patients (66.9%) and beta‐blockers in one‐third (32.0%) and more frequently in the ACS‐HF patients. In contrast, the use of spironolactone and digoxin was low (approximately 20% of patients).

Coronary angiography was undertaken in 75.2% and 46.5% of patients with ACS‐HF or CS, respectively. However, of the 2035 patients with ST‐elevation myocardial infarction (STEMI) (ACS‐HF [*n* = 1812] and CS [*n* = 223]), only 448 (24.7%) and 58 (26.0%), respectively, received reperfusion therapy with primary percutaneous coronary intervention (*Table* *3*).

### Early (first 24 h) coronary care unit management

Overall, the use of intravenous diuretics during the first 24 h was 76.6%, predominantly in those with PO (83.5%), HT‐HF (86.4%), and DHF (83.1%). In fact, almost all patients with CS (95.3%) received vasopressors. In contrast, approximately half of the patients with ACS‐HF (41.1%) and HT‐HF (47.5%) received intravenous vasodilators. Unexpectedly, early use of ACEIs or ARBs occurred in only one‐half of patients, while beta‐blockers or spironolactone were administered to only one‐tenth (*Table* *4*).

**TABLE 4:** Early treatment within 24 h of admission according to the clinical phenotypes

	Overall (*n* = 7759)	ACS‐HF (*n* = 3338)	DHF (*n* = 2617)	HT‐HF (*n* = 914)	CS (*n* = 404)	PO (*n* = 255)	RHF (*n* = 231)	*P* value
Intravenous diuretics (%)	76.6	71.4	83.1	86.4	56.2	83.5	66.2	<0.0001
Inotropes any (%)	13.0	8.7	11.3	6.2	66.1	25.9	15.6	<0.0001
Dobutamine (%)	10.3	7.5	7.4	3.7	59.2	21.6	12.6	<0.0001
Levosimendan (%)	0.8	0.7	0.6	0.2	4.5	0.8	0.0	<0.0001
Dopamine (%)	3.5	1.6	4.3	3.0	12.4	7.5	4.3	<0.0001
Vasopressors any (%)	14.8	9.8	9.8	3.6	95.3	37.6	20.8	<0.0001
Norepinephrine (%)	14.5	9.6	9.6	3.5	94.6	36.5	19.9	<0.0001
Vasopressin (%)	6.5	3.8	3.1	1.1	55.4	16.5	9.1	<0.0001
Both vasopressors (%)	6.2	3.6	2.8	1.0	54.7	15.3	8.2	<0.0001
Intravenous vasodilators any (%)	30.9	41.1	19.0	47.5	2.5	27.1	7.8	<0.0001
Nitroglycerine (%)	28.9	40.8	16.3	41.7	2.2	18.4	7.4	<0.0001
Nitroprusside (%)	2.3	0.4	2.8	6.9	0.2	9.0	0.4	<0.0001
ACEI (%)	50.2	63.0	44.3	53.7	5.7	25.1	25.1	<0.0001
ARBs (%)	1.7	0.8	2.6	3.3	0.0	0.4	1.7	<0.0001
ACEI or ARB (%)	51.7	63.7	46.5	56.3	5.7	25.5	26.8	<0.0001
Beta‐blocker (%)	11.1	14.7	10.0	9.7	1.5	3.5	3.0	<0.0001
Spironolactone (%)	9.1	5.6	15.1	8.4	2.0	9.4	7.8	<0.0001
Digoxin (%)	13.9	2.8	25.1	20.6	7.2	27.1	17.7	<0.0001
Amioradone (%)	7.1	4.6	9.5	7.1	12.6	6.7	7.8	<0.0001

ACEIs, angiotensin‐converting enzyme inhibitors; ACS‐HF, acute coronary syndrome and HF; ARBs, angiotensin receptor blockers; CS, cardiogenic shock; DHF, decompensation heart failure; HT‐HF, hypertensive HF; PO, pulmonary oedema; RHF, isolated right HF.

### Outcomes during hospitalization

Overall, the median hospital stay was 9 days (IQR 5–17 days), while the length of stay in the CCU was 5 days (IQR 3–7 days). In‐hospital mortality occurred in 1390 (17.9%) of the 7759 patients with AHF included in analyses. For the 13 283 patients without HF admitted to the same CCU during the study period, the following numbers and pathologies were recorded: ACS 59.5% (*n* = 7901), valvular heart disease 8.6% (*n* = 1140), cardiomyopathy 8.9% (*n* = 1182), lung disease 2.5% (*n* = 332), cardiac arrhythmia and atrial‐ventricular block 9.7% (*n* = 1292), pericardial disease 1.1% (*n* = 142), cardiac tumour 0.7% (*n* = 93), acute aortic syndrome 3.1% (*n* = 410), hypertensive crisis 0.7% (*n* = 94), adult congenital heart disease 1.6% (*n* = 214), and critically ill patients without cardiovascular disease 3.6% (*n* = 483). All‐cause in‐hospital mortality was higher among patients with AHF compared with patients without AHF (17.9% vs. 5.0%; *P* < 0.0001).

**Figure 3:**
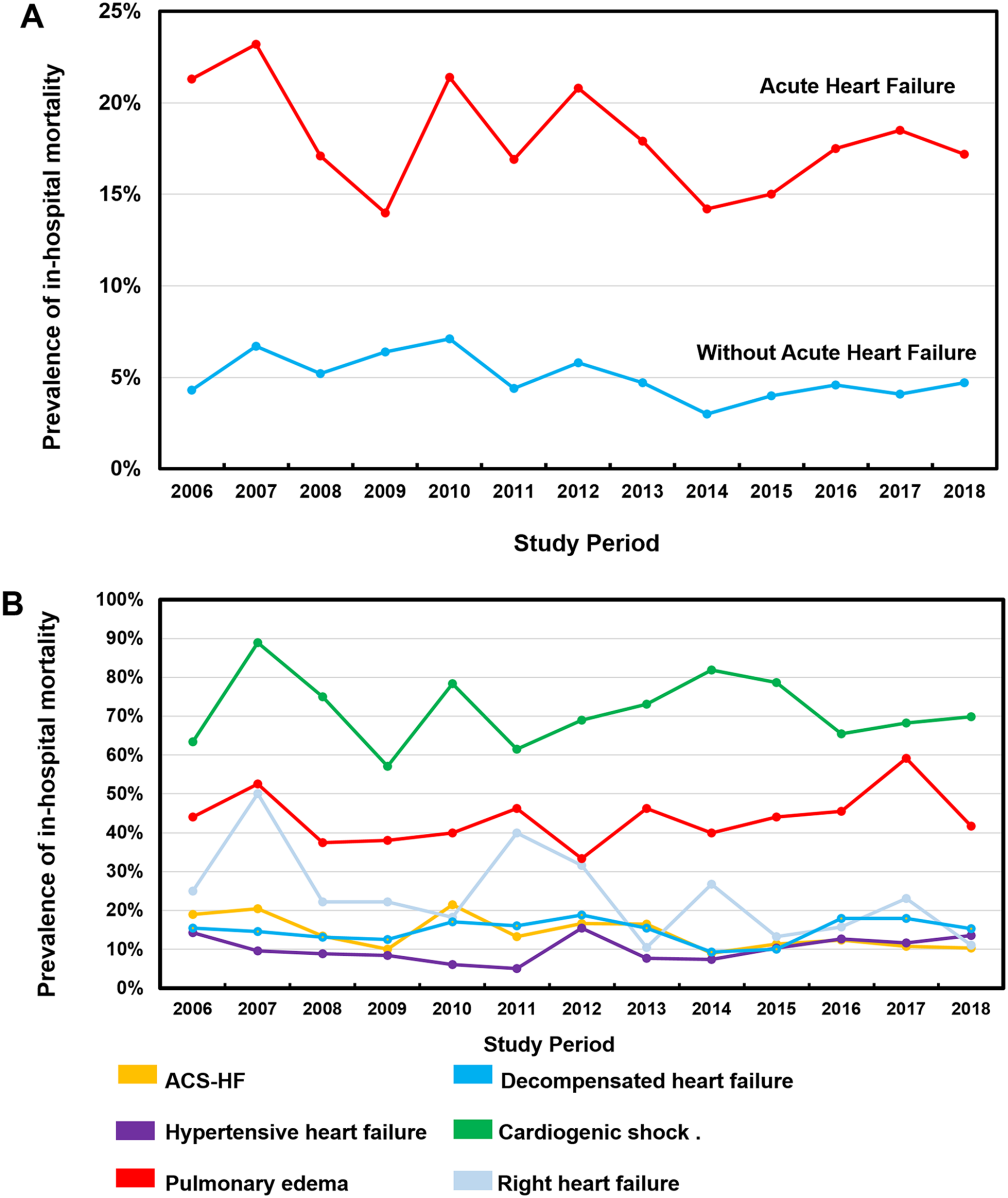
(A) Temporal trends from 2006 to 2018 in rates of all‐cause in‐hospital mortality in patients with and without acute heart failure hospitalized in the same coronary care unit during the same study period. (B) All‐cause in‐hospital mortality according to clinical phenotype of acute heart failure at admission. ACS‐HF, acute heart failure and associated acute coronary syndromes.

**Figure 4:**
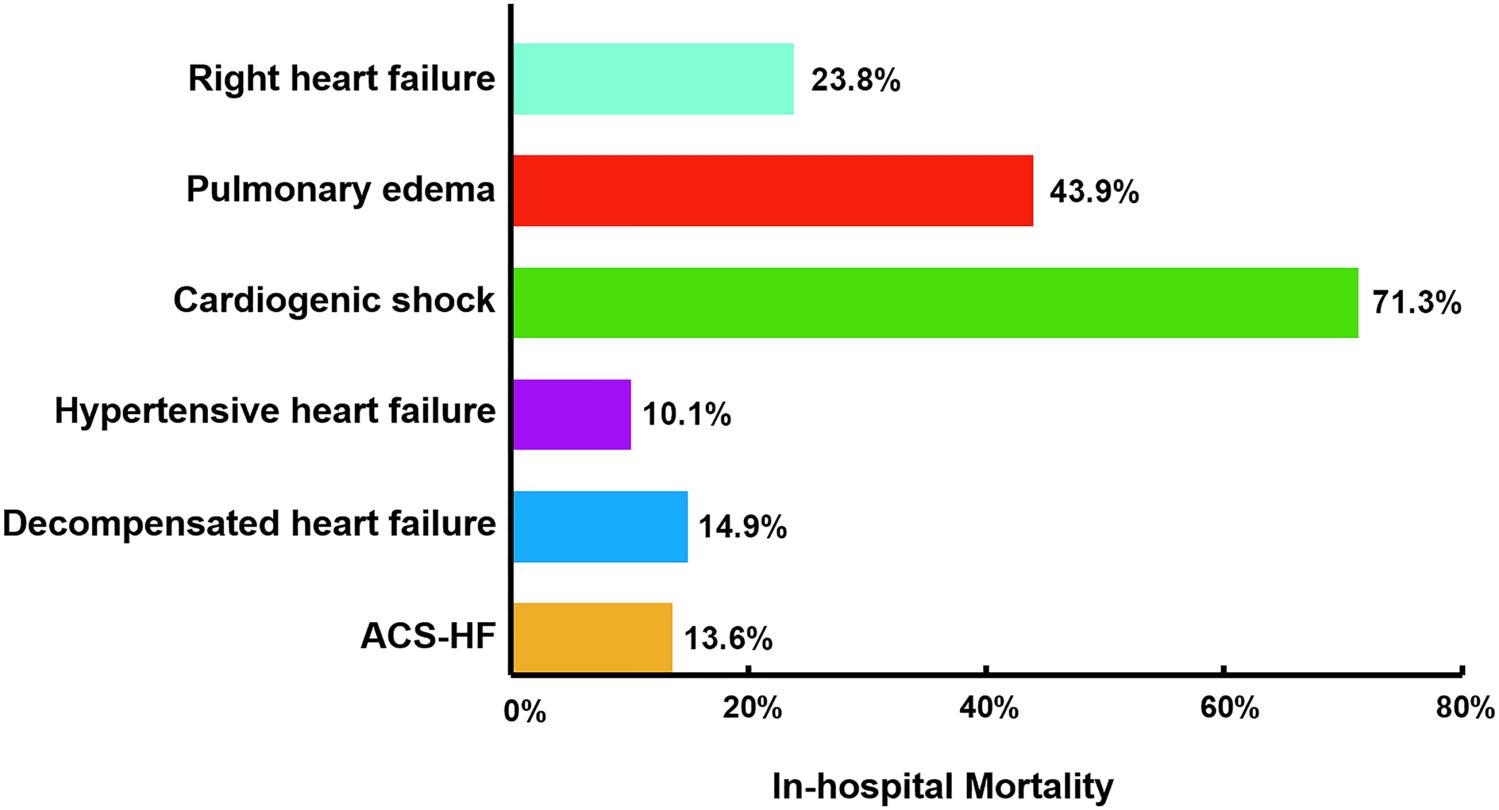
All‐cause in‐hospital mortality according to clinical phenotype of acute heart failure at admission. ACS‐HF, acute heart failure and associated acute coronary syndromes.

**Figure 5:**
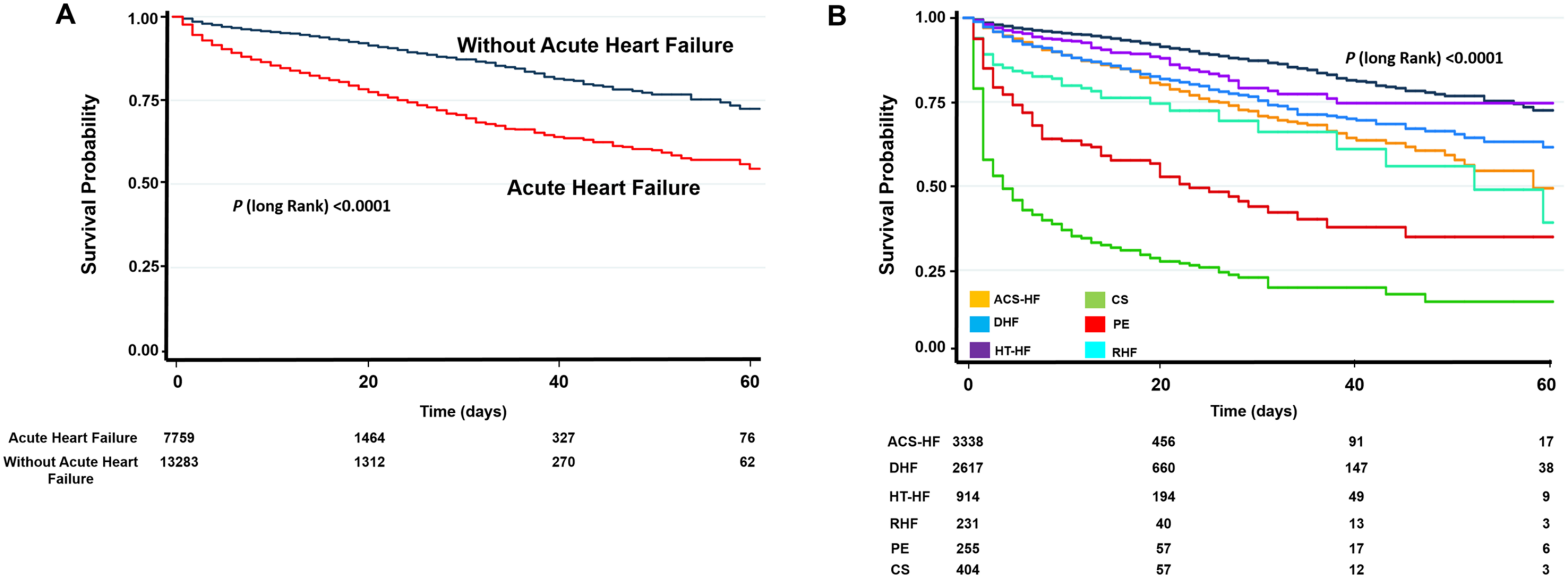
Kaplan–Meier curves for all‐cause in‐hospital mortality: (A) divided into hospitalized reference patients without heart failure (blue line) and patients with acute heart failure (red line); (B) using pairwise comparisons with reference patients without acute heart failure, there were differences in in‐hospital survival according to AHF clinical phenotype. ACS‐HF, acute heart failure and associated acute coronary syndromes; CS, cardiogenic shock; DHF, decompensated heart failure; HT‐HF, hypertensive heart failure; PO, pulmonary oedema; RHF, right heart failure.

Over the 13‐year period between 2006 and 2018, in‐hospital mortality decreased for patients with AHF (from 21.3% to 17.2%, *P*_trend_ = 0.01) (*Figure* *3A*). The unadjusted in‐hospital mortality rates were significantly higher in patients with CS (71.3%), followed by PO (43.9%), RHF (23.8%), DHF (14.9%), ACS‐HF (13.6%), and HT‐HF (10.1%) (*Figure* *4*). The mortality rates according to the year of presentation and the AHF phenotype are shown in *Figure* *3B*. Only the group of patients with ACS‐HF exhibited a decline in in‐hospital mortality between 2006 and 2018 (19.0 to 10.4%, *P*_trend_ < 0.0001). Using the patient group without HF as a reference in the age‐adjusted and gender‐adjusted Cox proportional hazards model, patients admitted with either CS or PO clinical phenotype showed a higher in‐hospital mortality risk (HR 16.6, 95% CI 14.48–19.19, *P* < 0.0001 and HR 7.4, 95% CI 6.05–9.08, *P* < 0.0001, respectively). At the other end of this spectrum, patients with HT‐HF or ADH clinical phenotypes showed a slightly higher risk of death compared with patients without HF (HR 1.4, 95% CI 1.18–1.83, *P* < 0.0001 and HR 1.42, 95% CI 1.30–1.56, *P* < 0.0001, respectively). Patients with AHF precipitated by ACS or RHF showed an intermediate risk of death compared with patients without HF (HR 2.3, 95% CI 2.09–2.66, *P* < 0.0001 and RHF HR 3.8, 95% CI 2.88–5.01, *P* < 0.0001, respectively) (*Figure* *5*).

Adjusted multivariate Cox proportional hazards regression models were generated with all significant univariate predictors of in‐hospital mortality listed in Supporting Information, *Table S* *2*. Using HT‐HF patients as a reference for the lowest in‐hospital mortality rate, the multivariate Cox proportional hazards analysis (Model 1) identified the clinical phenotypes of PO (HR 2.68, 95% CI 1.73–4.14, *P* < 0.0001) and CS (HR 3.37, 95% CI 2.12–5.35, *P* < 0.0001) as independent predictors of in‐hospital mortality among patients with AHF. Other factors independently associated with increased in‐hospital mortality were being female (*P* = 0.006), age per 10‐year group (*P* < 0.0001), previous valvular surgery (*P* = 0.01), *de novo* AHF (*P* = 0.005), sodium < 136 mEq/L (*P* = 0.001), hs‐CRP ≥ 10 mg/L (*P* < 0.0001), albumin < 3.5 g/dL (*P* = 0.03), eGFR ≤ 30 mL/min (*P* < 0.0001), and LVEF < 40% (*P* < 0.0001) (*Table* *5*).

**TABLE 5:** Multivariate analysis for the prediction of in‐hospital all‐cause mortality in patients with acute heart failure

Model 1	Hazard ratio	95% confidence interval	*P* value
Clinical phenotypes			
HT‐HF	Reference group		
ACS‐HF	1.01	0.83 to 1.45	0.95
DHF	0.98	0.66 to 1.45	0.92
RHF	1.28	0.75 to 2.20	0.35
PO	2.68	1.73 to 4.14	<0.0001
CS	3.37	2.12 to 5.35	<0.0001
Gender (female)	1.22	1.05 to 1.41	0.006
Age (per 10 years)	1.11	1.06 to 1.16	<0.0001
Previous smoking	0.80	0.69 to 0.93	0.005
Previous valvular surgery	1.31	1.05 to 1.62	0.01
*De novo* AHF	1.29	1.08 to 1.54	0.005
SBP ≥ 140 (mmHg) (%)	Reference group		
SBP 90–140 (mmHg) (%)	1.47	1.10 to 1.97	0.008
SBP < 90 (mmHg) (%)	1.62	1.11 to 2.37	0.01
Sodium < 136 mEq/L	1.24	1.09 to 1.41	0.001
hs‐CRP, ≥10 mg/L	1.57	1.32 to 1.87	<0.0001
Albumin, <3.5 g/dL	1.16	1.01 to 1.33	0.03
eGFR, ≤30 mL/min	1.59	1.36 to 1.85	<0.0001
LVEF ≥ 50%	Reference group		
LVEF 40–49%	0.86	0.70 to 1.04	0.17
LVEF < 40%	1.51	1.27 to 1.79	<0.0001
**Model 2**			
Clinical phenotypes			
HT‐HF	Reference group		
ACS‐HF	1.15	0.87 to 1.53	0.30
DHF	1.13	0.86 to 1.48	0.38
RHF	1.31	0.82 to 2.07	0.24
PO	1.93	1.38 to 2.71	<0.0001
CS	1.50	1.09 to 2.07	0.01
Gender (female)	1.17	1.01 to 1.36	0.02
Age (per 10 years)	1.13	1.08 to 1.19	<0.0001
Previous smoking	0.82	0.70 to 0.95	0.01
Previous valvular surgery	1.26	1.01 to 1.57	0.03
Sodium < 136 mEq/L	1.26	1.11 to 1.43	0.001
hs‐CRP, ≥10 mg/L	1.30	1.10 to 1.55	0.003
eGFR, ≤30 mL/min	1.47	1.21 to 1.72	<0.0001
LVEF ≥ 50%	Reference group		
LVEF 40–49%	0.90	0.73 to 1.11	0.35
LVEF < 40%	1.24	1.04 to 1.47	0.01
Intra‐aortic balloon pump	1.32	1.08 to 1.62	0.006
Mechanical ventilation*	1.80	1.452to 2.14	<0.0001
Intravenous diuretics**	0.70	0.59 to 0.84	<0.0001
Inotropes^***^	1.49	1.27 to 1.76	<0.0001
Vasopressors^****^	2.91	2.41 to 3.51	<0.0001

ACS‐HF, acute heart failure and associated acute coronary syndromes; AHF, acute heart failure; CS, cardiogenic shock; DHF, decompensated heart failure; eGFR, estimated glomerular filtration rate (according to the Cockroft–Gault formula); hs‐CRP, high‐sensitivity C‐reactive protein; HT‐HF, hypertensive heart failure; LVEF, left ventricular ejection fraction; MI, myocardial infarction; PCI, percutaneous coronary intervention; PO, pulmonary oedema; RHF, right heart failure; SBP, systolic blood pressure.

* Interaction with cardiogenic shock (*P* value for interaction < 0.0001) and with PO (*P* value for interaction = 0.001).

** Interaction with cardiogenic shock (*P* value for interaction < 0.0001), PO (*P* value for interaction < 0.0001), RHF (*P* value for interaction = 0.001), and with DHF (*P* value for interaction = 0.01).

^***^ Interaction with cardiogenic shock (*P* value for interaction < 0.0001).

^****^ Interaction with cardiogenic shock (*P* value for interaction < 0.0001) and with PO (*P* value for interaction = 0.04).

When the in‐hospital management was added to the multivariate model (Model 2), the use of inotropes (*P* < 0.0001), vasopressors (*P* < 0.0001), IABP (*P* < 0.0001), and mechanical ventilation (*P* < 0.0001) was independently associated with increased in‐hospital mortality. Conversely, the use of intravenous diuretics (*P* < 0.0001) was associated with a reduced risk of mortality. In Model 2, significant interactions were found between use of mechanical ventilation, inotropes, vasopressors, intravenous diuretics, and the clinical phenotypes of AHF, mainly PO and CS (*Table* *5*).

## Discussion

In this single‐centre cohort of patients with AHF admitted to a CCU in a centre specializing in cardiovascular diseases, we found a high mortality in patients with AHF, which may depend, among other factors, upon the AHF clinical phenotypes. We also found significant differences in clinical characteristics at presentation and in the aetiologies, compared with the large registries in Europe and the USA.

Reported prevalence rates of patients hospitalized with AHF have varied widely, both between and within countries (10–51%),^12^, ^13^ encompassing the prevalence of AHF in our sample (36.6%). Marked differences at hospital admission have been well documented across geographic regions, in terms of baseline demographics, clinical profiles, laboratory tests, comorbidity burden, and use of guideline‐recommended therapies.^14^, ^15^ To our knowledge, our study is the first to describe a large cohort of patients with AHF presenting to a hospital in Latin America who were classified by clinical phenotype.^16^ In contrast to previous studies,^17^ our sample's predominant clinical phenotype was AHF in an ACS context, while only one‐third had DHF. Chioncel *et al*.^18^ recently analysed 6629 patients with AHF from 21 European and Mediterranean countries who were enrolled in the ESC Heart Failure Long‐Term (ESC‐HF‐LT) registry. The authors found that the most frequent clinical phenotype was DHF (61.1%), followed by ACS‐HF (14.4%) and PO (13.2%).

There are notable differences in the characteristics of our sample of patients with AHF compared with their counterparts in the large registries from the USA and Europe.^8^, ^19^, ^20^, ^21^ Our sample of patients with AHF was younger and had lower prevalence rates of female sex, hypertension, diabetes, and previous atrial fibrillation. Nevertheless, their prevalence of *de novo* AHF and WCHF were comparable with those reported previously (45.2% and 54.8%, respectively).^22^ However, in our sample with WCHF, there was a striking underutilization of pre‐hospitalization evidence‐based medical therapies that improve outcomes and reduce the burden of hospitalization in patients with HF (i.e. diuretics, beta‐blockers, ACEIs/ARBs, and mineralocorticoid/aldosterone receptor antagonists).^3^ The use of ACEI/ARB in addition to a beta‐blocker is recommended for patients with HF and reduced LVEF to reduce the risk of HF hospitalization and death.^3^ This does not appear to be the case for the population with WCHF and reduced LVEF in our study because about one‐third were treated before hospitalization with the combination of ACEI/ARB and a beta‐blocker. These findings are surprising given that other research has shown that in patients with chronic HF with reduced LVEF, ACEIs/ARBs, beta‐blockers, and mineralocorticoid receptor antagonists were used in 92.2%, 92.7%, and 67.0% of patients, respectively.^23^

Overall, in our study, 81.4% of patients had pulmonary congestion and 39.6% had peripheral oedema; these percentages are similar to those reported from a recent registry (74.6% and 55%, respectively).^24^ Similarly, in our patients with RHF, the presence of pulmonary congestion (42.9%) was similar to that found in the study by Chioncel *et al*. (53.2%).^18^ Ventricular interdependence, increased intravascular volume, and changes in pulmonary lymphatic drainage have been postulated as the potential mechanisms to explain pulmonary congestion in RHF.^25^, ^26^

It is important to note that appropriate therapy requires early identification of the patient's specific clinical AHF phenotype. In our study, the use of evidence‐based medicines was adequate based on expert consensus‐based recommendations.^27^ Overall, the use of intravenous therapies such as diuretics (81%), vasodilators (31%), and inotropes (18.9%) was similar to that recently reported for hospitalized patients in the ESC‐HF‐LT registry.^18^ In addition, the present study shows that 76.6% of these patients were treated with intravenous diuretics within 24 h of admission, predominantly in those with PO (83.5%), a strategy that has been associated with lower in‐hospital mortality.^28^ Our study also found greater use of inotropes and vasopressors in patients with HT‐HF than in other studies (8.6% vs. 1.5%).^18^ This may reflect the inappropriate application of aggressive therapies for some types of AHF, for example, the combination of intravenous diuretics and intravenous vasodilators, which were given to half of our patients with HT‐HF.^29^ An outcome of this practice may have been iatrogenic hypotension and, as a consequence, the unjustified use of vasopressors and/or inotropes. Unfortunately, their use in patients with AHF has increased despite the evidence of their association with an increase in in‐hospital mortality.^30^, ^31^, ^32^

Investigators have recently shown that natriuretic peptide‐guided therapy facilitates the optimization of therapy in AHF patients and reduces the in‐hospital mortality. However, there is insufficient information about whether natriuretic peptide‐guided therapy can be applied routinely to all AHF patients.^33^ Unfortunately, in our study, it was not possible to evaluate the influence of NT pro‐BNP levels on in‐hospital treatment in each of the AHF phenotypes.

Coronary heart disease, valvular heart disease, and dilated cardiomyopathy are specific AHF‐associated cardiovascular conditions.^3^ Our study emphasizes the heterogeneity of AHF patients; coronary heart disease and valvular heart disease were the most frequent underlying diseases, occurring in 78% of patients with AHF, followed by cardiomyopathies. Of note was the distribution of underlying disease within each AHF clinical phenotype, as well as the overlap of aetiologies among them. Specifically, we found that 10% of our study sample had an aetiology that has been infrequently reported in previous studies, including congenital heart disease in adults, acute aortic syndromes, intracardiac tumours, and pericardial diseases.

Latin America‐based data are mainly from South America (usually Brazil and Argentina). The aetiology of AHF reported in this region is like other regions (i.e. coronary heart disease, valvular heart disease, and cardiomyopathy), with special interest in chagasic cardiomyopathy.^16^

Our patients' median hospital stay was 9 days, which is similar to that reported from European registries but longer than that reported in US registries.^19^, ^22^ Yet the most striking finding from the present study is the substantial rate of in‐hospital mortality (17.9%), which is in stark contrast to rates reported from European registries (6.4–7.3%) and in the USA (4%),^9^, ^19^ although it is similar to the overall mortality rate reported from intensive care units in a survey of 666 hospitals in nine countries (17.8%).^21^ However, other studies from Latin America have also reported high in‐hospital mortality (11.7%), with a higher rate among patients with a reduced ejection fraction, ischaemic heart disease, or Chagas disease.^34^

In our study population, after adjusting for all variables at admission as well as for treatment procedures, we found that the clinical phenotypes of PO and CS were independent predictors of in‐hospital mortality. This finding is consistent with that of Oliva *et al*., who showed that the phenotypes of CS and PO are independent predictors of in‐hospital mortality.^22^ Consistent with the literature, our study found that other factors associated with hospital mortality were renal dysfunction, older age, low systolic blood pressure, hyponatraemia, and low LVEF.^19^, ^22^, ^35^ An interesting finding is that the use of intravenous diuretics was associated with lower in‐hospital mortality rate, as has been shown in recent studies.^28^ By contrast, our analysis showed that intravenous inotrope and/or vasopressor use was an independent predictor of a detrimental outcome, which is consistent with the finding of previous studies.^30^, ^31^, ^32^

Several factors may explain the high rate of mortality in our sample, among which the predominant ACS clinical phenotype stands out. Previous data have shown that ACS complicated by AHF carries a particularly high risk of adverse outcomes, including the highest risk of short‐term death (around 13%).^36^, ^37^ Furthermore, the frequency of CS in our sample was higher compared with previous studies (5.2% vs. 3%) with a significantly higher in‐hospital mortality compared with that reported in the literature.^38^ In our population of patients with CS, ACS was the most common cause in most patients (69.1%), and other aetiologies were associated with the remaining 30%. This finding is consistent with that of Harjola *et al*.,^39^ who reported that 81% of the CS patients had ACS.

One interesting finding of our study was the high rate of in‐hospital mortality among patients with CS (69%), which contrasts with the published rate of 40%, depending on the underlying aetiology.^38^ It is now well established from a variety of studies that the prognosis of patients with acute myocardial infarction (AMI) complicated by CS has improved over the past decade mainly thanks to early revascularization.^40^, ^41^ Primary percutaneous coronary intervention was only performed in a quarter of the STEMI patients with CS, suggesting that the majority of patients with STEMI delayed their hospital attendance.

On the other hand, in our analysis, the IABP was used only on 25.7% of all patients with CS. Data from several studies suggest that IABP is now less and less often used, and on the contrary, the application of other mechanical circulatory support has increased both in Europe and in the USA.^42^, ^43^, ^44^ In our CCU, IABP is the most widely used mechanical circulatory support device because other advanced forms of mechanical circulatory support are not available for us yet.

The Heart Failure Association of the ESC has suggested that, despite advances in therapy, CS remains the most common cause of in‐hospital death after AMI and is a major cause of death in young patients with other potentially reversible underlying cardiac pathologies. According to the Heart Failure Association, CS management should consider appropriate organization of the health‐care services, and therapies must be given to appropriately selected patients in a timely manner, while avoiding iatrogenic harm. This association also suggested that further research is needed on this topic.^45^

Alternatively, in our sample of patients with WCHF, HF therapies that modify the disease (e.g. ACEIs, ARBs, beta‐blockers, and mineralocorticoid/aldosterone receptor antagonists) were underutilized.^3^ Finally, patients with endocarditis and prosthetic dysfunction were included in the group with valvular heart disease; both of these in the presence of AHF are associated with a high rate of in‐hospital mortality.^46^, ^47^

Analysis of HF patients from lower‐income to middle‐income and high‐inequality countries has showed higher mortality rates than those patients from high‐income and low‐inequality countries.^48^ In developing countries such as Mexico, unfavourable social circumstances, along with inadequate and inefficient public spending on health care, can present considerable barriers to improving outcomes in patients with AHF.^49^ There is a need to develop more practical strategies to improve adherence to guidelines. Such strategies should be based on multidisciplinary models involving HF teams, structured referral schemes, telemedicine, synchronized education of patients and health‐care providers, care standardization, and quality control and auditing. The development of centres of excellence, such as those recently described for the treatment of advanced HF, may contribute to this goal.^50^

### Study limitations

Our study has several limitations. First, our retrospective data reflect the experiences of a single tertiary university centre specialized in cardiovascular diseases. Therefore, we cannot be certain that these cases represent the overall AHF patient population in Mexico. Second, these patients were not classified according to the 2008 ESC guidelines at hospital admission; rather, we applied this classification retrospectively for the purposes of these analyses and may potentially have incomplete or inaccurate results. Third, the absence of criteria for the degree of severity in pulmonary congestion may turn out to be an erroneous classification that overlaps between patients with PO and HT‐HF. Finally, our study included only patients who were hospitalized in the CCU, rather than including those hospitalized in internal wards as was the case in other reports.

## Conclusions

These data, from real‐world AHF patients admitted to a CCU of a university hospital in a developing Latin American country, show significant differences in both clinical characteristics at presentation and aetiologies compared with large European and US registries. The present study highlights a high in‐hospital mortality rate that may reflect a high‐risk patient cohort and/or inefficient public spending on health care for patients with chronic HF. Regardless, this profile represents a significant public health challenge.

## Conflict of interest

None declared.

## Supporting information


**Table S1.** Medications at hospital admission according to the clinical phenotypes.
**Table S2.** Univariate analysis for the prediction of in‐hospital all‐cause mortality in patients with acute heart failure.
