# Supplementary material for: Clinical phenotypes, aetiologies, management, and mortality in acute heart failure: a single‐institution study in Latin‐America
Source: ESC Heart Fail. 2020 Nov 11;8(1):423–37. doi: 10.1002/ehf2.13092 (PMC7835571; doi:10.1002/ehf2.13092)
Supplement: Supplementary file 1 — Table S1. Medications at hospital admission according to the clinical phenotypes. Table S2. Univariate analysis for the prediction of in‐hospital all‐cause mortality in patients with acute heart failure. [file EHF2-8-423-s001.docx]

**Table 1S. Medications at hospital admission according to the clinical phenotypes (Available data in 7,355 patients)**

|  | | | | | | | | |
| --- | --- | --- | --- | --- | --- | --- | --- | --- |
|  | **Overall**  **(n = 4,402)** | **ACS-HF**  **(n = 890)** | **DHF (n = 2,444)** | **HT-HF (n = 646)** | **CS (n = 124)** | **PE (n = 172)** | **RHF (n = 126)** | ***P-* Value** |
| **ACEI, %** | 42.5 | 51.0 | 40.5 | 41.6 | 50.0 | 36.6 | 28.6 | <0.0001 |
| **ARBs, %** | 13.7 | 16.0 | 11.3 | 20.9 | 16.1 | 12.2 | 5.6 | <0.0001 |
| **ACEI or ARBs, %** | 55.4 | 66.2 | 51.1 | 61.3 | 65.3 | 48.8 | 33.3 | <0.0001 |
| **Beta-Blocker, %** | 38.4 | 52.1 | 34.6 | 35.6 | 56.5 | 33.7 | 18.3 | <0.0001 |
| **Diuretics, %** | 54.2 | 42.5 | 57.5 | 53.4 | 62.1 | 64.0 | 54.8 | <0.0001 |
| **Spironolactone, %** | 28.8 | 19.2 | 32.2 | 20.9 | 46.8 | 43.0 | 33.3 | <0.0001 |
| **Digoxin, %** | 26.4 | 13.1 | 30.7 | 22.4 | 39.5 | 37.8 | 28.6 | <0.0001 |
| **Oral nitrate, %** | 9.6 | 24.9 | 5.3 | 8.5 | 5.6 | 4.1 | 2.4 | <0.0001 |
| **Amioradone, %** | 6.9 | 4.2 | 7.7 | 6.3 | 13.7 | 11.0 | 2.4 | <0.0001 |
| ***De novo* heart failure** | | | | | | | | |
|  | **Overall**  **(n = 2,953)** | **ACS-HF**  **(n = 2,321)** | **DHF (n = 0)** | **HT-HF (n = 239)** | **CS (n = 248)** | **PO (n = 56)** | **RHF (n = 89)** | ***P-* Value** |
| **ACEI, %** | 26.9 | 27.5 | -- | 32.2 | 23.4 | 21.4 | 10.1 | <0.0001 |
| **ARBs, %** | 14.1 | 14.1 | -- | 20.1 | 8.9 | 10.7 | 13.5 | 0.01 |
| **ACEI or ARBs, %** | 40.4 | 41.1 | --- | 50.6 | 32.3 | 32.1 | 23.6 | <0.0001 |
| **Beta-Blocker, %** | 18.2 | 17.5 | --- | 25.1 | 15.7 | 19.6 | 21.3 | 0.04 |
| **Diuretics, %** | 11.9 | 9.7 | --- | 25.5 | 11.3 | 25.0 | 28.1 | <0.0001 |
| **Spironolactone, %** | 2.6 | 1.8 | --- | 7.5 | 2.4 | 8.9 | 7.9 | <0.0001 |
| **Digoxin, %** | 2.3 | 1.3 | --- | 8.8 | 2.4 | 5.4 | 9.0 | <0.0001 |
| **Oral nitrate, %** | 6.0 | 6.6 | --- | 5.9 | 3.2 | 0.0 | 2.2 | 0.03 |
| **Amioradone, %** | 0.7 | 0.5 | --- | 1.7 | 0.8 | 1.8 | 4.5 | <0.0001 |

ACS-HF, Acute coronary syndrome and HF; DHF, Decompensation heart failure; HT-HF, Hypertensive HF; CS, Cardiogenic shock; PO, Pulmonary oedema; RHF, Isolated right HF;

ACEI, angiotensin-converting enzyme inhibitors; ARBs, angiotensin receptor blockers.

**Table 2S. Univariate analysis for the prediction of in-hospital all-cause mortality in patients with acute heart failure.**

|  | **Hazard ratio** | **95% Confidence Interval** | **P Value** |
| --- | --- | --- | --- |
| **HT-HF** | **Reference group** | | |
| **ACS-HF** | 1.50 | 1.19 to 1.87 | <0.0001 |
| **DHF** | 1.41 | 1.12 to 1.77 | 0.003 |
| **RHF** | 2.48 | 1.78 to 3.47 | <0.0001 |
| **PE** | 4.55 | 3.45 to 6.00 | <0.0001 |
| **CS** | 10.17 | 8.04 to 12.87 | <0.0001 |
| **Gender (Female)** | 1.24 | 1.12 to 1.38 | <0.0001 |
| **Age (per 10years)** | 1.08 | 1.04 to 1.12 | <0.0001 |
| **Body mass index, (per kg/m^2^)** | 0.98 | 0.97 to 0.99 | 0.007 |
| **Current smoking** | 1.15 | 1.00 to 1.32 | 0.04 |
| **Previous smoking** | 0.77 | 0.69 to 0.87 | <0.0001 |
| **Dyslipidemia** | 0.84 | 0.74 to 0.95 | 0.007 |
| **Diabetes** | 1.08 | 0.97 to 1.20 | 0.15 |
| **Previous heart failure** | 0.71 | 0.64 to 0.80 | <0.0001 |
| **Previous MI** | 0.85 | 0.74 to 0.97 | 0.02 |
| **Previous PCI** | 0.77 | 0.62 to 0.94 | 0.01 |
| **Previous valvular surgery** | 1.22 | 1.03 to 1.44 | 0.01 |
| **Previous AF** | 1.12 | 0.98 to 1.29 | 0.08 |
| **Novo AHF** | 1.45 | 1.31 to 1.62 | <0.0001 |
|  |  |  |  |
| **SBP >140, mmHg, (%)** | **Reference group** | | |
| **SBP <90, mmHg, (%)** | 6.64 | 5.49 to 8.03 | <0.0001 |
| **SBP 90-140, mmHg, (%)** | 1.70 | 1.44 to 2.01 | <0.0001 |
| **Sodium < 136 mEq/L** | 1.43 | 1.29 to 1.59 | <0.0001 |
| **Albumin, <3.5 g/dL** | 1.68 | 1.48 to 1.90 | <0.0001 |
| **hs-CRP, (per mg/L),** | 1.001 | 1.001 to 1.001 | <0.0001 |
| **hs-CRP, ≥10 mg/L** | 2.00 | 1.71 to 2.35 | <0.0001 |
| **eGFR, ≤ 30 ml/min** | 2.14 | 1.91 to 2.40 | <0.0001 |
|  |  |  |  |
| **LVEF ≥ 50%** | **Reference group** | | |
| **LVEF 40-49%** | 0.77 | 0.65 to 0.92 | 0.005 |
| **LVEF < 40%** | 1.45 | 1.28 to 1.63 | <0.0001 |
| **Intra-aortic balloon pump** | 3.42 | 2.95 to 3.93 | <0.0001 |
| **Mechanical ventilation** | 6.00 | 5.43 to 6.70 | <0.0001 |
| **Intravenous diuretics** | 0.56 | 0.50 to 0.63 | <0.0001 |
| **Inotropes** | 4.15 | 3.74 to 4.62 | <0.0001 |
| **Vasopressors** | 7.87 | 7.05 to 8.79 | <0.0001 |
| **Intravenous vasodilators** | 0.61 | 0.54 to 0.69 | <0.0001 |

HT-HF, hypertensive heart failure; ACS-HF, acute heart failure and associated acute coronary syndromes; DHF, decompensated heart failure; RHF, right heart failure; PE, pulmonary edema; CS, cardiogenic shock; MI, myocardial infarction; PCI, percutaneous coronary intervention; AF, atrial fibrillation; AHF, acute heart failure; SBP, Systolic blood pressure; hs-CRP, high-sensitivity C-reactive protein; eGFR, estimated glomerular ﬁltration rate (according to the Cockroft-Gault formula), LVEF, left ventricular ejection fraction.
